# Allelic diversity of the Vrn genes and the control of growth habit and earliness in wheat

**DOI:** 10.18699/VJGB-23-108

**Published:** 2023-12

**Authors:** S.E. Smolenskaya, N.P. Goncharov

**Affiliations:** Institute of Cytology and Genetics of the Siberian Branch of the Russian Academy of Sciences, Novosibirsk, Russia; Institute of Cytology and Genetics of the Siberian Branch of the Russian Academy of Sciences, Novosibirsk, Russia

**Keywords:** wheat, Vrn genes, winter/spring growth habit, length of plant vegetative period, earliness, пшеница, гены Vrn, яровость, озимость, длина вегетационного периода, скороспелость

## Abstract

Wheat is one of three main food crops around the world, which has the largest distribution area due to its adaptation to the different environments. This review considers polymorphisms and allelic variation of the vernalization response genes Vrn controlling the major adaptation traits in wheats (the genus Triticum L.): growth habit (spring vs. winter) and length of vegetative period (earliness). The review summarizes available information on the allelic diversity of the Vrn genes and discusses molecular-level relationships between Vrn polymorphisms and their effect on growth habit (spring vs. winter) and earliness (length vegetative period in spring plants) in di-, tetra- and hexaploid wheat species. A unique attempt has been made to relate information on mutations (polymorphisms) in dominant Vrn alleles to the values of the commercially most important trait “length of plant vegetative period (earliness)”. The effects of mutations (polymorphisms) in the recessive vrn genes on vernalization requirement in winter wheats are considered, and this trait was formalized. The evolution of the winter/spring growth habit in the genus Triticum species is discussed. A scheme of phylogenetic interactions between Vrn alleles was constructed on the basis of these polymorphisms; the paper considers the possibilities to enhance the diversity of polymorphisms for the dominant Vrn genes and their alleles using wheat related species and rarely used alleles and discusses the prospects of breeding for improved earliness for concrete agroecological zones.

## Abstract (ru)

Пшеница, являясь одной из трех основных продовольственных культур мира, занимает самый ши- рокий ареал за счет адаптивности к разнообразным условиям возделывания. В обзоре рассматриваются поли- морфизм и аллельная изменчивость генов Vrn (от англ. response to vernalization), контролирующих важнейшие адаптационные признаки пшениц – тип (яровость vs. озимость) и скорость развития у диких и возделываемых видов пшениц (род Triticum L.). Суммируется информация об аллельном разнообразии генов Vrn и обсуждается связь полиморфизмов этих генов на молекулярном уровне с их влиянием не только на признак «тип развития (яровость vs. озимость)», но и на признак «скороспелость (длина вегетационного периода яровых растений, ДВП)» у ди-, тетра- и гексаплоидных видов. Предпринята попытка связать полученную информацию о мута- циях (полиморфизмах) доминантных аллелей генов Vrn с выраженностью наиболее важного с хозяйственной точки зрения признака «продолжительность ДВП (скороспелость)», которая ранее в обзорах не предприни- малась. Рассматривается влияние мутаций (полиморфизмов) в последовательностях рецессивных генов vrn на признак «потребность в яровизации» у озимых форм растений пшениц и выполнена его формализация. Обсуждается эволюция озимости/яровости в роде Triticum. На основе выявленных полиморфизмов построена схема филогенетических взаимодействий аллелей генов Vrn и рассматриваются возможности расширения по- лиморфизма по доминантным генам Vrn и их аллелям за счет видов-сородичей и редко используемых алелей и перспективы селекции на изменение ДВП (скороспелости) для конкретных зон возделывания

## Introduction

Many of the cultivated and wild herbaceous plant species growing in temperate climates have developed the spring growth habit (cryophobic plants) or the winter growth habit (cryophilic plants) as adaptations to natural environments (Gupalo, Skripchinsky, 1971). The spring plants complete their entire development cycle during a single vegetation season, while the winter ones do not proceed to reproduction unless they have been exposed to low temperatures

In wild and cultivated wheat species, delays in transition from vegetative to reproductive development are controlled by the vernalization response genes Vrn regulating growth habit (spring vs. winter) and earliness, the vernalization requirement duration genes Vrd controlling duration of vernalization treatment in winter wheats, and the photoperiod sensitivity/insensitivity gene Ppd for response to photoperiod. Any of the dominant genes Vrn: Vrn-1 (Yan et al., 2003), Vrn-3 (Yan et al., 2006) and Vrn-D4 (Kippes et al., 2016), controls spring growth habit and is epistatic over the recessive alleles of these genes. An exception is the dominant gene Vrn2 described in Triticum monococcum L. (Yan et al., 2004a) and controlling winter growth habit: this gene expression are destroyed in polyploid wheat species.

An any dominant allele of the gene Vrn, except Vrn2, is enough for a plant to become a spring (Pugsley, 1971; Yan et al., 2004b; Fu et al., 2005; Knippes et al., 2018). Winter hexaploid wheat varieties are homozygous for the recessive alleles of all the three Vrn-1 genes at once (Stelmakh, 1987); while winter tetraploids, for two genes, Vrn-A1 and Vrn-B1, because the dominant genes Vrn-3 and Vrn-D4 have no recessive alleles, and so all winter varieties carry their null-alleles. No interaction between Vrd and Vrn has been described. A number of investigations have proposed schemes for the interaction between the Vrn with Ppd (Chen A. et al., 2014). However, the mechanisms underlying the interactions between these genes are not yet fully understood (Goncharov, 2012; Kiseleva, Salina, 2018). The Vrn genes are estimated to account for about 75 % of the control of variability of the trait duration of the vegetative period (DVP), and the Ppd genes, for about 20 % (Stelmakh, 1981). The third group of loci, EPS (earliness per se), controlling earliness per se, is under polygenic control (van Beem et al., 2005; Royo et al., 2020) and explains only about 5 % of DVP variation (Stelmakh, 1981).

The Vrn-1 controlling the adaptability of wheat to environments (the traits spring/winter growth habit and earliness) are transcription factor genes (Trevaskis et al., 2003; Yan et al., 2003) that determine the expression of many genes involved in response to environmental stresses. Mutations in such genes not only disrupt their function, but also cause remarkable phenotypic changes. In wheat, DVP (earliness) is one of the important traits allowing the wild and cultivated species to take full advantage of the springsummer season. At the same time, the Vrn genes have direct effects on plant productivity, yield and resistance to stresses, such as drought, low temperatures, pests and diseases, to mention a few (Zotova et al., 2019).

This paper considers the results of the modern molecular and genetic studies concerning spring/winter growth habit control and the effect of the allelic diversity of the Vrn genes on DVP in spring plants.

## How many VRN loci does wheat have?

To date, six dominant Vrn genes (three Vrn-1: Vrn-A1, Vrn- B1, and Vrn-D1) (Yan et al., 2003), two Vrn-3 (Vrn-A3, and Vrn-B3) (Nishimura et al., 2018), one Vrn-D4 (Kippes et al., 2016)) and one recessive gene, vrn-2 (Yan et al., 2004a) have been described as the ones controlling spring growth habit. Let us consider their main features

VRN-1 locus. In di-, tetra- and hexaploid wheats, the spring growth habit is most commonly controlled by the Vrn-1 genes (Genotypes…, 1985; Catalogue…, 1987; Goncharov, 1998; Lysenko et al., 2014; among others). These genes are located in distal part of long arms of the homeologous group 5 chromosomes: Vrn-A1 on 5AL (Law et al., 1976; Galiba et al., 1995; Dubcovsky et al., 1998), Vrn-B1 on 5BL (Barrett et al., 2002; Iwaki et al., 2002) and Vrn-D1 on 5DL (Law et al., 1976). It has been shown that the Vrn-1 genes are orthologous to the Arabidopsis thaliana (L.) Heynh. closely related CAULIFLOWER (CAL), APETALA1 (AP1) and FURITFULL (FUL) meristem identity genes controlling the reproductive/flowering meristem transition (Ferrándiz et al., 2000; Yan et al., 2003; Preston, Kellogg, 2006; Dhillon et al., 2010). As was found, in Arabidopsis, FUL controls not only the development of carpels and fruits, but also flowering time (Ferrándiz et al., 2000). Later, another gene, WAP1 (Wheat APETALA1) were characterized as AP1-like MADS-box gene in common wheat), was found and characterized as an activator of the transition from vegetative to reproductive development (Yan et al., 2003). It was shown that WAP1 in wheat corresponds to Vrn-1 (Trevaskis et al., 2003). WAP1 expression begins before the transition to reproductive phases and continues until maturity (Murai et al., 2003).

The dominant Vrn-A1 alleles have insertions and/or deletions in the promoter regions as well insertions and/or deletions and single nucleotide polymorphisms (SNPs) in the first intron, which the native recessive gene vrn-A1 does not (Supplementary Material)1. Deletions in the first intron is what differentiates most of the dominant Vrn-B1 alleles differ from the recessive vrn-B1 allele. Additionally, deletions or insertions within the first intron are features of the dominant Vrn-D1 alleles.


Supplementary Materials are available in the online version of the paper: https://vavilov.elpub.ru/jour/manager/files/Suppl_Smolen_Engl_27_8.pdf


Although molecular biological methods allowed a large number of alleles of the dominant Vrn genes to be described (Yan et al., 2004a, b; Fu et al., 2005; Liu et al., 2012; Milec et al., 2023), the effects of these alleles on the duration of vernalization treatment and flowering time were not always identified (see Supplementary Material).

VRN-2 locus. The Vrn2 gene (Vrn-Am2) has been revealed only in the diploidic wheat T. monococcum (Dubcovsky et al., 1998). This gene was mapped to the distal part of the long arm of chromosome 5Am within the segment translocated from 4AmS (Dubcovsky et al., 2006). The VRN-2 locus includes two completely linked zinc finger-CCT domain genes ZCCT1 and ZCCT2 that act as flowering repressors down-regulated by vernalization (Yan et al., 2004a). However, it was established that the main determinant for Vrn-2 expression in diploid wheat T. monococcum and T. boeoticum Boiss. and barley Hordeum vulgare L is day length (Dubcovsky et al., 2006; Trevaskis et al., 2006).

The sequence of the Vrn-2 genes was revealed in the winter common wheat Jagger and 2174. No allelic variants of Vrn-A2 in the A genome or Vrn-D2 in the D genome were found (Chen Y. et al., 2009). Two duplicated copies of Vrn-B2 were found in 2174. The Vrn-B2 allele was not found in Jagger, suggesting this variety carries a null allele of this gene. The null-allele had no effect on flowering time in a segregated population. Mapping of Vrn-B2 showed that both of its copies in 2174 were closely associated with a SNP on chromosome 4BL, suggesting that the Vrn-B2 duplicates were located in a tandem-like manner at the same locus. Identical Vrn-B2 sequences have been found in contig sequences of chromosomes 4BS, 2BS and 5DL in Chinese Spring (CS) (International Wheat Genome Sequencing Consortium..., 2018). In Aegilops squarrosa L. (=syn. Ae. tauschii Coss.), the sequence of the gene Vrn-D2 was not found (Chepurnov et al., 2023). Thus, the gene Vrn-2 in tetra- and hexaploid wheats is inactivated (Tan, Yan, 2016).

VRN-3 locus. The dominant gene Vrn-B3 (formerly Vrn 5 or Vrn-B4) was mapped to the short arm of chromosome 7B using 82 recombinants obtained from crosses between CS and the substitution line CS/Hope 7B (Yan et al., 2006). The gene is activated by vernalization and long day; it has been identified as an orthologue of the gene FLOWERING LOCUS T (FT ) in Arabidopsis (Yan et al., 2006; Cockram et al., 2007). It is not easy to understand the role of TaFT in flowering regulation, because both common wheat and barley each possesses a 78 % identical paralogous copy of FT2 (TaFT2 and HvFT2, respectively) (Yan et al., 2006; Faure et al., 2007). As the TaFT/TaFT2 duplication event took place after these cereals and Arabidopsis split off, this event is unrelated to the duplication of FT/TSF, the twin sister of FT found in Arabidopsis (Li, Dubcovsky, 2008).

The dominant gene Vrn-A3 (homologous to Vrn-B3) has only been revealed in tetraploid wheats and mapped on the short arm of chromosome 7A (Nishimura et al., 2018). It is unlikely that it has homologs in common wheat, and it must be inactive as is vrn-2 in T. monococcum.

VRN-4 locus. The dominant gene Vrn-D4 was discovered in the line Gabo-2 (Knott, 1959; Pugsley, 1972; Goncharov, 2003) selected from the Australian commercial common wheat cv. Gabo. This gene was localized on chromosome 5D (Kato, 1993) and mapped to the centromeric region of the same chromosome (Kippes et al., 2015). The most current hypothesis is that the dominant gene Vrn-D4 can have emerged in polyploid wheats due to a translocation of a ~290 kb-fragment of the long arm of chromosomes 5A to the proximal part of the short arm of chromosome 5D (Kippes et al., 2015). The translocated segment includes a Vrn-A1 copy that carries mutations in the coding and regulatory regions (Kippes et al., 2015).

The gene is expressed at earlier stages of spring plants,(See scales for growth and development in cereals (Efremova, Chumanova, 2023). and its sequence does not contain any of mutations that were previously described for the dominant gene Vrn-A1 and that endow common wheat with spring growth habit (Yan et al., 2003, 2004b). The dominant gene Vrn-D4 has instead three SNPs in the first intron, where the binding site for the TaGRP2 protein described as a negative regulator for Vrn-A1 is located (Fu et al., 2005).

At present, no B-genome genes homologous to Vrn-4 are known. As the dominant gene Vrn-D4 has not been found in Ae. tauschii, the D-genome donor to hexaploid wheat (Chepurnov et al., 2023), it can be concluded that this mutation occurred in polyploids.

Thus, spring wheat carry mutations in the promoter or the first intron of the Vrn genes (Yan et al., 2004b; Fu et al., 2005). At the same time, most of the dominant alleles of the Vrn-1 genes described to date (Vrn-A1a, Vrn-Am1a, Vrn-A1b, Vrn-A1d, Vrn-A1e, Vrn-Am1g, Vrn-A1h and Vrn- A1i) carry mutations in the promoter regions, within the VRN box, including SNPs, indels or its full elimination (Shcherban, Salina, 2017). The mutations found in the Vrn genes are presented in Supplementary Material. Chromosomal locations of the Vrn genes are detailed in Table 1. They were confirmed by molecular biological studies (Kiseleva, Salina, 2018).

**Table 1.**
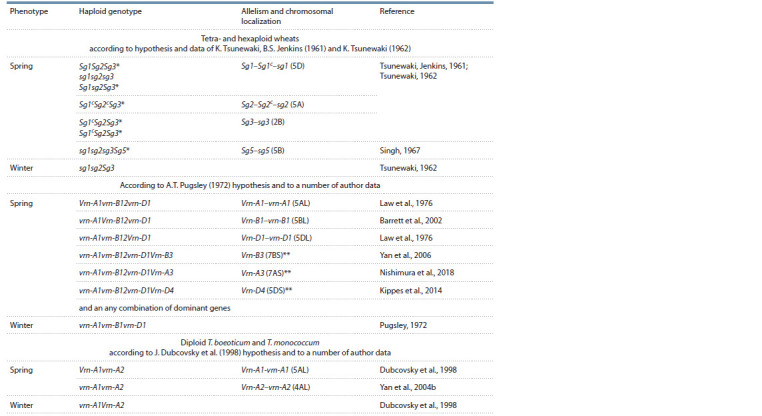
Designation and localization in chromosomes of genes for growth habit in spring and winter wheat (after Goncharov (2012) with addition) * Spring growth habit is observed for any allelic state of the gene Sg3. ** The gene does not have recessive alleles.

The fact that the dominant alleles of the Vrn-1 genes carry insertions and deletions that the recessive (intact) alleles do not may be an indication that they are evolutionarily younger (Milec et al., 2023). This allows their phylogenetic relationships to be inferred (see the Figure).

**Fig. 1.**
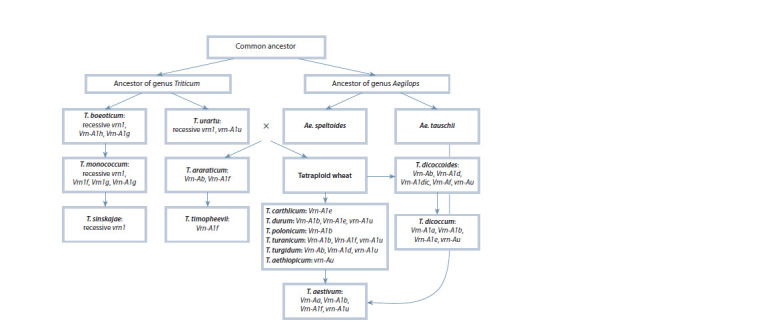
Scheme of Triticum and Aegilops genera evolution (according to Goncharov, 2011, with additions). Different alleles of Vrn-A1 gene among wheat species are presented in appropriate boxes next to the species names. Section Timopheeevii is presented in grey boxes, while section Monococcon, Dicoccoides and Triticum are in white ones (after Konopatskaia et al., 2016, with additions).

## Vernalization of winter and spring wheats and its molecular and genetic network

Vernalization is the need of winter plants adapted to temperate climates for exposure to low temperatures, ensuring the transition of them from vegetative to reproductive development. A requirement for vernalization is an adaptive trait that helps prevent flowering before winter and permits flowering in the favorable conditions of spring. Winter plants are assumed to carry recessive (native) alleles of thevrn genes, with mutations in any of them leading to partial or complete inhibition of response to vernalization (Fu et al., 2005; Milec et al., 2023) and to a conversion of winter growth habit to spring ones. Spring plants form ears without vernalization, even though late-ripening spring forms, including the facultative growth ones (Facultative growth habit is an agrotechnological characteristic. Facultative growth habit plants can be both autumn-sowing and spring-sowing as reserve crops. At present, the State Register of RF includes three facultative growth habit cultivars produced in the Lukyanenko National Grain Center (Krasnodar, Russia) (State Register…, 2023).), may respond to vernalization by promoting earliness and a reducing DVP. Vernalization in the late-ripening spring plants is poorly studied. In southern latitudes, vernalization is believed to provide autumn-sown late-ripening spring plants protection against damage from early-autumn light frosts. 

A major obstacle to the study of the transition from vegetative to reproductive development is misidentification of the functions performed by the alleles of the Vrn genes. The misidentification arose from a terminological confusion started by Australian scientists A.T. Pugsley (1968) and R.A. McIntosh (1973), who were unfortunate to replace “spring growth” (Tsunewaki, 1962) with “response to vernalization” (Pugsley, 1971) (see Table 1). This term replacement were certain reasons (Pugsley, 1968); however, they were rather speculative. Years went by, but even so J. Dubcovsky, a molecular biologist, overlooked the issue and allowed this terminological mess to become part of the subsequent editions of “Catalogue of Gene Symbols for Wheat” (McIntosh et al., 2013). Note that the gene symbol Sg (spring growth) has immediate relevance to the trait spring growth habit vs. winter growth habit and allows this trait to be explicitly formalized (Goncharov, 2004). In this case, the classification of the trait is genotype-based, not phenotype-based (Steinfort et al., 2017).

Need to pay attention that genotyping and phenotyping data may be inconsistent (see Table 2 in M. Makhoul (2022)). This relates to autumn-sown spring cultivars in the southern regions of the Eastern Hemisphere (Makhoul et al., 2022). Unfortunately, it is becoming more and more popular to state (postulated) the phenotypes depending on the sowing season (Steinfort et al., 2017). While, the phenotyping has to base solely on growth habit of plant (spring vs. winter). A.T. Pugsley (1983) begins his terminologyrelated considerations with “winter growth habit”, that is, the physiological condition of a wheat plant requiring treatment to low temperatures (vernalization) and, consequently, having “response to vernalization” before it can come to reproductive phase.

**Table 2.**
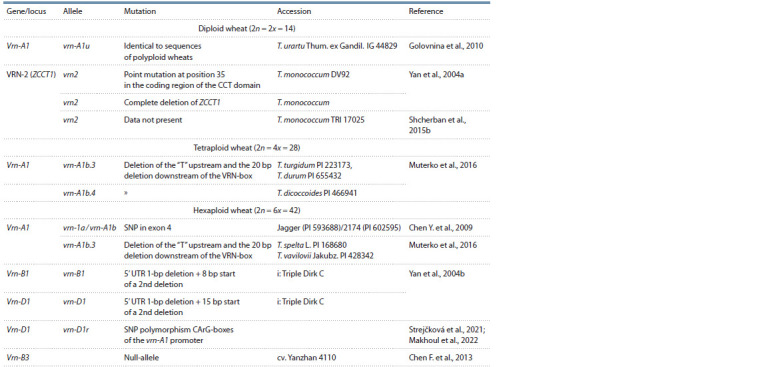
Polymorphism of recessive alleles of the Vrn genes in winter wheat Mutations in the recessive alleles of the gene vrn-A1 in hexaploid wheat (Chen Y. et al., 2009) and the gene Vrn-2 in diploidic wheat (Yan et al., 2004b) are in the coding regions.

And only the next step (question) is about phenotyping based on growth habit (spring vs. winter). The trait has to phenotyped as a qualitative morphological one (Goncharov, 2004). Plant phenotypes differ in that some plants switch to reproductive growth within a single spring-summer season and some do not. Wheat varieties are phenotyped with respect to this trait in the summer, at high positive temperatures, during ontogenesis after planting in the field or a greenhouse.

Response to vernalization is a quantitative trait, and so the accessions should be phenotyped using low temperatures (vernalization). In this case, the ultimate question is one about the duration of vernalization treatment (Dolgushin, 1935). Spring plants, even late-ripening ones, do not require vernalization to proceed to reproductive growth. Producing a unified approach for phenotyping spring/winter will make it possible to correctly compare all available research results

In the database Wheat Trait Ontology, the traits plant growth habit (vernalization) and earliness are in the same subclass Development of class Trai and are associated with plant phenotype (Nédellec et al., 2020). The trait response to vernalization is not there, it is in the subclass Response to environmental conditions, meaning are the response of plants to the influence of the external environment (to a stress factor).

An important part of a unified approach to defining and phenotyping a trait is not only the terminology, but also the symbols of wheat genes. After the power to decide was shifted from one group of researchers (Ausemus et al., 1946) to another (McIntosh et al., 1973), the misleading terms “response to vernalization” became “legalized”.

That is why, although Vrn is a legal abbreviation and lateripening spring and facultative (intermediate) growth habit varieties have response to vernalization, we will be using a more relevant term “growth habit (spring vs. winter)” throughout. We suggest the term “vernalization response” be left only for winter wheat (Fayt et al., 2018). Whether or not the recessive genes vrn really control vernalization response in winter varieties is still a question. Let us have a closer look at the matter.

## Polymorphism of the recessive alleles of Vrn genes in winter wheat

All dominant Vrn genes known to date that control the qualitative difference between spring and winter wheats have been cloned. Two mutually exclusive hypotheses have been proposed: one stating that the duration of vernalization treatment in winter plants depends on the variability of the recessive alleles vrn-A1 for winter growth habit (Pugsley, 1971; Chen Y. et al., 2009, 2010) and the other stating that it depends on a system of genes independent of them (Gotoh, 1979; Bulavka, 1984; Fayt, 2003, 2006a, b; Stelmakh et al., 2005) and unrelated to the expression of the recessive vrn genes.

This process has been poorly studied genetically and not studied at all at the molecular and biological level. Now it is obvious that the polymorphisms for the recessive genes vrn in winter wheat varieties do not explain differences in the duration of vernalization treatment between these varieties (Table 2). Not a single exception invalidating this genetic model has been reported in the studies, in which a large number of cultivars/germplasms from wheat species with different ploidy levels were screened using molecular markers for the recessive alleles of the each of Vrn-1 genes (Yan et al., 2003, 2004a, b; Fu et al., 2005; Bonnin et al., 2008; Zhang X.K. et al., 2008; Santra et al., 2009; Chen Y. et al., 2010). The polymorphism for the Vrn-3B and Vrn-4D genes, in which the recessive allele is represented only by only as a null allele, makes an exception.

The alleles that have SNPs in exon 4 of the recessive gene vrn-A1 are associated with the regulation of the development of winter plants and are designated vrn-A1a in Jagger (PI 593688) and vrn-A1b in 2174 (PI 602595). In a field assessment of a population of 96 recombinant inbred lines from crosses between Jagger and 2174, Y. Chen et al. (2009) showed that the vrn-A1a plants had an earlier onset of shooting. At the same time, the effects of the alleles on the duration of vernalization treatment have not been checked experimentally

Plants with the 3_SNPs haplotype showed higher transcription levels of the gene Vrn-A1 than 1_SNP plants (Kippes et al., 2018). An assumption was made that the single nucleotide polymorphism in the regulatory region of the first intron should probably be associated with differences in the duration of vernalization treatment in the winter wheat. However, the attempt made by N. Kippes et al. (2018) to associate the SNPs in the recessive gene vrn-A1 with the duration of vernalization treatment cannot be recognized successful: the authors used the winter near-isogenic line Triple Dirk C, a derivative of the spring cv. Triple Dirk, and in our experiments, Triple Dirk C plants, in the field, progressed into shooting (and some came to ear) within four months without vernalization (Goncharov, 2012).

It can be concluded that none of the known changes (point mutations) in the sequences of the recessive genes vrn has any effect on the duration of vernalization treatment in the winter wheat accessions (see Table 2).

Note that isogenic lines for the Vrd genes controlling variation in the duration of vernalization treatment in winter common wheat have long since been created (Fayt, 2006b) and can now be used in molecular and biological experiments. These genes reside on the winter common wheat’s chromosomes: Vrd1 on 4A and Vrd2 on 5D (Fayt et al., 2007).

Allelic variability at the VRN locus and winter growth habit. Mutations in the regulatory regions of the Vrn-1 gene are associated with prevalent spring growth habit, while the point mutations of a gene (or genes) at the VRN-2 locus (the vrn-2a allele) or the deletion of an entire gene (the vrn-2b allele) are also associated with spring growth as a recessive trait in diploid wheat T. monococcum and barley H. vulgare (Yan et al., 2004b; Dubcovsky et al., 2005). No multiple allelism of the dominant gene Vrn-2 controlling winter growth habit has been revealed. This offers indirect evidence that this gene is not associated with the duration of vernalization treatment in the diploid T. monococcum or T. boeoticum. What genes control it at barley is not known either.

## Variability of dominant alleles of the Vrn genes in spring accessions of di-, tetra- and hexaploid wheats and their effect on duration of the vegetative period

The number of works analyzing the distribution of the dominant genes Vrn and their alleles in the main wheat cultivation areas is impressive (Catalogue…, 1987; Goncharov, 1998; Fu et al., 2005; Zhang X.K. et al., 2008; Lysenko et al., 2014; Smolenskaya et al., 2022; and others). Differences of the regions by alleles is shown (Genotypes…, 1987; Stelmakh, 1990; Goncharov, 1998). As far as modern spring common wheat are concerned, Vrn-A1a is prevalent in cold-winter areas where spring wheat are sown only in the spring. By contrast, the dominant alleles of the homologous genes Vrn-B1a and Vrn-D1a are highly frequent in the varieties cultivated in the Mediterranean climate, where spring wheats are sown in the autumn (Stelmakh, 1990; Zhang X.K. et al., 2008; Shcherban, et al., 2015a). Noteworthy, Vrn-D1a emerged in Southern Europe in the 1930s together with photoperiod-insensitivity and reduced height genes coming from Japanese common wheat (Goncharov, 2012). The question as to whether the dominant gene Vrn-B3 can be widely used outside China (Bonnin et al., 2008) requires special close consideration. This gene has not been found in Russia’s cultivars (Lysenko et al., 2014), nor has it been found in the progeny of the variety Hope (Goncharov, Gaidalenok, 2005), the gene Vrn-B3 donor for the isogenic line CS/Hope 7B.

Facultative growth habit plants. In English-language literature, facultative growth plants are known as “intermediate” (Flood, Halloran, 1986). According to B.V. Rigin and the colleagues, the spring growth habit in them should be determined by the dominant Vrn-A1 gene (Genotypes…, 1985), while in A.F. Stelmakh’ opinion, exclusively by the dominant gene Vrn-B1 (Stelmakh, 1981). In Chinese wheat, the facultative growth habit plants possess the dominant allele Vrn-D1b (Zhang X.K. et al., 2008).

Because facultative growth habit plants (sometimes called semi-spring) play an important role in wheat production in some areas (Fayt et al., 2018), 689 Chinese varieties were studied for the frequency and distribution of the allele Vrn-D1b in them. The results showed that allele Vrn-D1a, Vrn-D1b and vrn-D1 were present in 27.3, 20.6 and 52.1 % of the specimens, respectively. Pedigree analysis indicates that Vrn-D1b originated from Chinese landraces (Guo et al., 2015)

A study of F2 hybrid segregating for Vrn-D1b and Vrn- D1a in greenhouse long-day conditions without vernalization showed that the Vrn-D1b homozygote plants would heading 32 days later than Vrn-D1a homozygotes. Because Vrn-D1b has the same deletion in the first intron as does Vrn-D1a and a single nucleotide mutation in the promoter region and is associated with facultative growth habit, the authors proposed that the mutation in the promoter can change the basal activity level of gene Vrn-D1, which is already active due to the deletion in the first intron (Zhang J. et al., 2012).

Copy number of the Vrn genes. Change in the copy number (CNV) of the Vrn-1 genes is one of the sources of genetic variability in hexaploid wheat (Díaz et al., 2012; Würschum et al., 2015). In most cases, CNV is associated with changes in gene Vrn expression (Muterko, 2023); however, data on their effect on the DVP are inconsistent.

## Hexaploid wheat species (2n = 6x = 42)

The most economically important point in the study of allelism of the dominant genes Vrn is the search for their functional association with the DVP. Data on DVP (earliness) in spring wheat are quite inconsistent. According to K.A. Flaksberger (1938), it is in a range between 76 and 140 or more days. Other authors report variations from 70–80 to 120–130 days (Kumakov, 1980). Opinions differ as to how to classify commercial common wheat varieties by maturity (Goncharov N.P., Goncharov P.L., 2018), as this classification has a clear-cut region-specific flavor. At the same time, earliness can be associated with different combinations of the dominant alleles of the Vrn genes (see Supplementary Material).

VRN-A1 allele. The distribution of spring common wheat into ripeness groups revealed that this trait is influenced by a combinations of certain dominant genes Vrn and their alleles (Stelmakh, 1993; Likhenko et al., 2014; Smolenskaya et al., 2022). Spring varieties with the dominant gene Vrn-A1 are usually more early-ripening than the varieties with dominant genes Vrn-B1 and Vrn-D1 (Stelmakh, 1993). It has been demonstrated the main contributor to the reduction in duration between emergence of plant seedlings and heading is the dominant allele Vrn-A1a, while Vrn-A1b, in contrast, accounts for later heading (Efremova et al., 2016). Additionally, the varieties with the dominant allele Vrn-A1b is rare in Siberia, 8 % (Smolenskaya et al., 2022). The Vrn-A1a has an insertion in promoter region and Vrn-A1b, in contrast, a deletion (Yan et al., 2004b).

B.V. Rigin and the colleagues (2021) stated that the ultra- ripening lines Rico (K-65588) and Rimax (K-67257) had the shortest time from emergence plant shootings to heading among all spring common wheat accessions in the VIR collection. Their genotypes revealed dominant alleles for three Vrn genes at once, Vrn-A1, Vrn-B1 (respectively Vrn-B1a or Vrn-B1c), and Vrn-D1.

Any of the dominant alleles, Vrn-A1a or Vrn-A1b, disables response to vernalization, while any of the dominant alleles of the Vrn-B1 or Vrn-D1 genes induces a residual response and leads to later flowering (Stelmakh, 1993). These data were confirmed by studies showing that the dominant alleles Vrn-A1a and Vrn-A1b in combination with the dominant gene Vrn-B1 can provide optimum flowering time and potentially high yield in the Pacific Northwest region of the USA, while spring wheat varieties with the dominant gene Vrn-D1 may have advantage in Idaho and Oregon, where the vegetation periods are longer (Santra et al., 2009).

VRN-B1 allele. A novel allele, Vrn-B1c, probably associated with earlier ripening in late-ripening spring varieties was revealed using near-isogenic lines with different alleles of the Vrn-B1 gene (Shcherban et al., 2012a). Its prevalence among common wheat varieties in Western Siberia and the North Kazakhstan, when spring growth habit being under monogenic control, was demonstrated (Shcherban et al., 2012b). In the absence of epistatic effects of the dominant Vrn-A1 gene, this allele causes earlier heading than does Vrn-B1a (Shcherban et al., 2013). The effect of Vrn-B1f on heading time is similar to that of Vrn-B1c, but the mechanism of its regulation most likely appears to be different (Strejčková et al., 2021).

VRN-D1 allele. The dominant gene Vrn-D1 occurs only in hexaploid wheat cultivars in the Asian region and some Italian varieties (Stelmakh, 1993; Goncharov, 1998). K. Iwaki and the colleagues (2000, 2001) found the dominant allele Vrn-D4 in a large number of common wheat cultivars from different regions worldwide (55 cultivars out of 272 studied). The highest frequency of occurrence was observed in accessions from India and the bordering countries (Iwaki et al., 2000, 2001). This dominant gene had previously been found in most accessions of the Indian hexaploid endemic species T. sphaerococcum Perciv. (Goncharov, Shitova, 1999).

The dominant allele Vrn-D t1 with a 5.4-kb deletion in the first intron was found in spring plants of Ae. tauschii from the Middle East (Takumi et al., 2011). One more dominant allele was described later (Chepurnov et al., 2023). This allele has effect on heading time.

All the variants identified in three Vrn-1 homeologs in wheat were designated as separate alleles, but not all of them were experimentally confirmed to have any effect on DVP (earliness) (see Supplementary Material).

VRN-B3 allele. The nucleotide substitutions or insertions/ deletions in the copies of the FT gene (Vrn-B3) in the A and D genomes in 239 local, old local and modern commercial cultivars from different regions cause DVP polymorphisms (Bonnin et al., 2008). In contrast to Vrn-1, the homeologous copies of the FT gene showed no evidence of epistatic interactions (Bonnin et al., 2008). TaFT overexpression in transgenic T. aestivum plants considerably accelerated flowering compared to the non-transgenic control (Yan et al., 2006).

The absence of isogenic lines does not allow its different alleles to be compared for the strength of their phenotypic manifestation. Note that, line 620 with Vrn-B3 had much later heading (Goncharov, 2012). Later heading was also observed in cultivars carrying various Vrn-B3 alleles, Vrn- B3a and Vrn-B3b (Chen F. et al., 2013), and Vrn-B3d and Vrn-B3e (Berezhnaya et al., 2021).

Two hundred and seventy eight Chinese spring common wheat cultivars were characterized using molecular markers of the Vrn-A1, Vrn-B1, Vrn-D1 and Vrn-B3 genes. The varieties possessing from three to four dominant Vrn genes, including the rare dominant gene Vrn-B3, were the earliest (30–31 days to heading on average), and one-, two-, or three-gene combinations, including the dominant gene Vrn-A1, but not Vrn-B3, followed (38 days to heading on average). On the basis of these data, the dominant Vrn-1 genes were ranked according to the amount of their influence on DVP reduction in the Chinese cultivars: Vrn-A1 > Vrn-B1 > Vrn-D1 (Zhang X.K. et al., 2008). This ranking is not the same as those reported elsewhere (Gotoh, 1979; Goncharov, 2003).

VRN-D4 allele. The dominant gene Vrn-D4 has a weaker effect on DVP than have the Vrn-A1, Vrn-D1 or Vrn-B3 genes (Kippes et al., 2014), but stronger than Vrn-B1 (Gotoh, 1979; Goncharov, 1998).

Tetraploid wheat species (2n = 4x = 28)

Based on the analysis of Vrn-A3 expression using sister lines, earlier flowering in accession TN26 of T. dicoccum Schrank ex Schuebl. than in accession TN28 of T. pyramidale Perciv. is due to a 7-bp insert in the promoter region of the gene which, this insert including a cis-element of the GATA box (Nishimura et al., 2018). The analysis revealed the presence of the early-flowering alleles of Vrn-A3 in spelt wheat from Ethiopia and India and their absence in the accessions of T. durum Desf. and common wheats. These results led the authors to the conclusion that the Vrn-A3a- h1 and Vrn-A3a-h2 alleles should be useful in breeding for earliness in durum and common wheat (Nishimura et al., 2021).

T. carthlicum Nevski and T. dicoccum accessions possessing the Vrn-B1c allele with a retrotransposon insertion passed to flower without vernalization. Transcripts in the winter DH-lines possessing the recessive vrn-B1 allele were observed no sooner than after vernalization (Chu et al., 2011).

Two spring accessions, PI 208912 (Iraq) of T. turgidum and PI 74830 (China) of T. durum and one winter accession PI 221422 (Serbia) of T. turgidum started to flower without vernalization. However, they did so substantially later than plants with the dominant Vrn-A1 or Vrn-B1 genes. Interestingly, winter accession PI 221422 started to flower 25 days later than did spring accessions PI 208912 and PI 74830. All of them have recessive vrn-B1 alleles and null alleles vrn-B3. It is proposed that their late flowering is due to the Vrn-A1i allele (Muterko et al., 2016).

The combination of the dominant Vrn-A1 and Vrn-B1s alleles was associated with early flowering the tetraploid species T. dicoccum and T. dicoccoides (Körn. ex Asch. et Graebn.) Schweinf. (Muterko et al., 2016). Vrn-A1 allelism is a possibility in T. dicoccum (Rigin et al., 1994).

The gene’s allelic variant coming from T. militinae Zhir. et Migusch. was designated Vrn-A1f-like. QTL analysis showed that it caused a 1.9–18.6-day delay in the flowering time of Tähti and Mooni, depending on cultivation conditions (Ivaničová et al., 2016).

In all T. timopheevii (Zhuk.) Zhuk. accessions studied, the spring growth habit was associated with the dominant Vrn-A1f-ins and Vrn-A1f-del/ins alleles (Golovnina et al., 2010; Shcherban et al., 2016). The same allele was found in wild T. araraticum Jakubz. (Golovnina et al., 2010). Noteworthy, this species has an extremely limited number of spring forms (Goncharov, 1998).

Diploid wheat species (2n = 2x = 14)

It is possible that the pattern of inheritance in diploid wheats is more sophisticated than it used to be thought before, as spring growth habit in the wild T. boeoticum has recently been shown to be under digenic control (Fu Hao, Boguslavskyi, 2023). Similar results obtained for the T. monococcum by L. Smith (1939) have remained unnoticed. Spring accessions of T. urartu, the Au-genome donor for polyploid wheat species, were found to have a Vrn-A1 mutation typical for the section Triticum species (Golovnina et al., 2009). However, as few as four T. urartu accessions from among 400 studied were spring (Goncharov, 1998), of which two were “odd” in that they were T. urartu phenotypically (with velvety pubescence of leaves), but T. boeoticum karyotypically (Adonina et al., 2015) and, therefore, Vrn-A1 polymorphism is most likely to have emerged no sooner than in polyploid wheats.

In field conditions, T. monococcum with various deletions in the promoter region of the Vrn-A1f and Vrn-A1g alleles showed 59–60 days to heading on average and did not differ significantly from each other in terms of this measure ( p = 0.842) (Chepurnov, Blinov, 2022).

## Enhancing the diversity of polymorphisms in the Vrn genes and prospects in breeding for reduced duration of the vegetative period

The polymorphism in dominant Vrn genes controlling spring growth habit in varieties of Siberia and the European part of Russian Federation is extremely low (Lysenko et al., 2014; Smolenskaya et al., 2022). In 75 % of the cultivars in Siberia, this trait is under digenic control exerted by the dominant Vrn-A1 and Vrn-B1 genes; in 25 %, under monogenic control exerted by dominant genes (among 24 cultivars, 19 are controlled by a single dominant gene Vrn-A1 and 5, by a single dominant gene Vrn-B1). Trigenic control was discovered for one cultivar, Tulun 15, (Likhenko et al., 2014). The conclusion made by E.A. Moiseeva and N.P. Goncharov (2007) that spring growth habit in the of Western and Eastern Siberian wheat cultivars is controlled by two dominant Vrn genes has been confirmed. An increased prevalence of the allele Vrn-B1c in West Siberian cultivars and of the allele Vrn-B1a in East Siberian cultivars has been observed, suggesting their selectivity to environments of these regions (Smolenskaya et al., 2022). Other regions of the Russian Federation have not yet been considered with this amount of scrutiny (Lysenko et al., 2014).

Our assumption is that, the level of DVP-related polymorphism in spring wheat cultivars in Siberia in particular and Russia in general can be enhanced by introgression of the dominant alleles of the Vrn genes from their wild ancestor (Goncharov, Chikida, 1995; Goncharov, 1998) or by using rare alleles that are present in their gene pool (Stelmakh, Avsenin, 1996; Koval, Goncharov, 1998) but have not been studied by a molecular genetic methods and are rarely used in the breeding. Note that T. urartu Thum. ex Gandil. – the donor of the Au genome of polyploid wheats does not carry any mutation that could be new to spring common wheat (Golovnina et al., 2010). The use of the diploid species T. monococcum carrying the Ab genome (Goncharov et al., 2007; Nishiura et al., 2018) appears to be impracticable either, due to its evolutionary unrelatedness to cultivated wheat. Consequently, the model based on T. monococcum is not successful, as it leads modern plant cultivation nowhere.

The aim of the future efforts is to develop a simple model predicting wheat phenology, with effects of vernalization and photoperiods taken into account. New facts about the expression of the Vrn genes, their allelic composition, and interaction with other genes will allow us to learn more about the associations known to date (Distelfeld et al., 2009; Jin, Wei, 2016; Krasileva et al., 2017; Kiseleva, Salina, 2018; Milec et al., 2023). This knowledge will undoubtedly contribute to increasing the efficiency of next generation breeding.

## Conflict of interest

The authors declare no conflict of interest.
